# Water droplet impact on elastic superhydrophobic surfaces

**DOI:** 10.1038/srep30328

**Published:** 2016-07-27

**Authors:** Patricia B. Weisensee, Junjiao Tian, Nenad Miljkovic, William P. King

**Affiliations:** 1 Department of Mechanical Science and Engineering, University of Illinois at Urbana-Champaign, 1206 W Green St., Urbana, IL, 61801, USA; 2 International Institute for Carbon Neutral Energy Research (WPI-I2CNER), Kyushu University, 744 Motooka, Nishi-ku, Fukuoka 819-0395, Japan

## Abstract

Water droplet impact on surfaces is a ubiquitous phenomenon in nature and industry, where the time of contact between droplet and surface influences the transfer of mass, momentum and energy. To manipulate and reduce the contact time of impacting droplets, previous publications report tailoring of surface microstructures that influence the droplet - surface interface. Here we show that surface elasticity also affects droplet impact, where a droplet impacting an elastic superhydrophobic surface can lead to a two-fold reduction in contact time compared to equivalent rigid surfaces. Using high speed imaging, we investigated the impact dynamics on elastic nanostructured superhydrophobic substrates having membrane and cantilever designs with stiffness 0.5–7630 N/m. Upon impact, the droplet excites the substrate to oscillate, while during liquid retraction, the substrate imparts vertical momentum back to the droplet with a springboard effect, causing early droplet lift-off with reduced contact time. Through detailed experimental and theoretical analysis, we show that this novel springboarding phenomenon is achieved for a specific range of Weber numbers (*We* >40) and droplet Froude numbers during spreading (*Fr* >1). The observation of the substrate elasticity-mediated droplet springboard effect provides new insight into droplet impact physics.

Dynamics of droplet impact are important in many natural processes^1^,^2^ and industrial applications, including anti-icing^3^,^4^, spray cooling^5^,^6^, pesticide and herbicide delivery^7^,^8^, and ink-jet printing^9^. Droplet impact is governed by the complex flow physics arising within the deforming droplet, manifesting itself in the form of droplet lateral spreading and recoil. As the droplet impacts the surface, its kinetic energy is re-directed in the lateral direction, flattening the droplet and converting the kinetic energy into surface energy. On low-friction surfaces, this kinetic-to-surface energy conversion process is very efficient, resulting in minimal energy dissipation due to viscous effects^10^. Once all of the kinetic energy has been converted to surface energy of the flattened droplet, the reverse surface-to-kinetic energy conversion process initiates, resulting in droplet retraction and lift off in the vertical direction. The total contact time from initial impact to lift-off, *t*_*c*_, influences the mass, momentum, and energy exchange between the droplet and the solid. Hence, achieving control of the contact time through manipulation of the internal flow physics dictates the transport processes occurring at the liquid-solid interface.

The contact time of water impact on a plane, rigid superhydrophobic surface is governed by the droplet size. Upon impact, the droplet spreads, reaches a maximum diameter, fully retracts, and vertically lifts off the surface^11^,^10^. For Weber numbers *We* = (*ρv*^*2*^*D*_*0*_)/*γ* >1, where *ρ*, *D*_*0*_, *v* and *γ* are the droplet density, initial diameter, impact speed, and surface tension, respectively, the droplet undergoes elastic impact. By balancing the droplet impact inertia (~*ρD*_*0*_/*t*_*c*_^2^) with capillarity (~*γ*/*D*_*0*_^2^), the contact time scales as *t*_*c*_ ~ (*ρD*_*0*_^3^/*γ*)^1/2^, and is independent of the impact speed^12^,^13^. Consequently, the only way to adjust the contact time on a plane, rigid, single length scale superhydrophobic surface is by controlling the droplet diameter.

One approach to reduce the contact time is to use a surface with hierarchical superhydrophobic surface features. Droplets impacting miniature superhydrophobic ridges can break impact symmetry and decrease the contact time, by influencing droplet deformation near the miniature features and initiating early de-wetting^1^,^14^. The same effect can be induced using a curved surface, when the curvature is on the order of the droplet diameter^15^. Also, during droplet recoil on hierarchical superhydrophobic posts, the surface-to-kinetic energy conversion can be enhanced by storing surface energy in the liquid between the miniature posts during impact, and recovering this energy during rebound^16^,^17^. For high speed impacts (*We* >12), droplets lift off near their maximum spreading diameter in a pancake-like shape, reducing their contact time by a factor of 4. These previous approaches require fabrication of miniature features on the impacted surface, and that the impacting droplet is precisely aligned with these features. These surface structuring approaches rely on the droplet to be the energy storage mechanism (surface energy) during impact and recoil. Taking inspiration from nature (leaves) and human technology (springboards), we study droplet impact dynamics on elastic superhydrophobic substrates as a passive mechanism for controlling and reducing contact time. We hypothesize that droplets impacting elastic surfaces might exhibit distinct dynamics, resulting in energy storage and recovery not just within the droplet but also within the elastic surface. By studying droplet impact on superhydrophobic elastic polymer sheets, we show that droplets can undergo springboarding and reduce contact times by a factor of 2 when compared to rigid superhydrophobic surfaces. Further experimental observation and theoretical analysis elucidates a surface mediated energy storage mechanism arising from the coupling of the substrate elastic response to impact, and the droplet internal flow dynamics. In contrast to previous studies, we show that droplet dynamics can be altered and contact times reduced by introducing a second energy storage mechanism during impact – elastic energy of the substrate – in addition to surface energy of the droplet. We present new fundamental knowledge of droplet impact physics and provide a starting point for more advanced approaches to enhance the performance of droplet-based applications by using substrate elasticity to achieve enhanced thermal, mass, or momentum transport.

## Results

### Impact Dynamics

**Figure 1:**
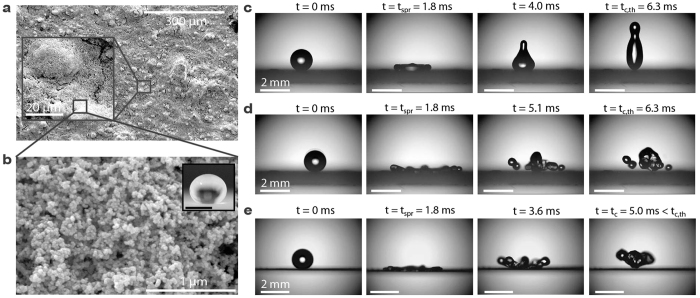
Surface characterization and dynamic behavior of water droplet impact on rigid and elastic superhydrophobic surfaces. (**a**) SEM micrographs showing the macroscale roughness of the NeverWet superhydrophobic coating due to particle clustering. (**b**) High resolution SEM micrograph showing individual nanoparticles. Inset: water droplet on a NeverWet coated glass slide (scale bar 1 mm). The advancing and receding contact angles were 164 ± 4° and 159 ± 3°, respectively. (**c**) High speed images showing a low velocity droplet (*v* = 0.68 m/s) impacting a rigid superhydrophobic surface, and following the classical model of spreading, retraction and lift-off at the theoretical contact time *t*_*c,th*_ = 2.6 (*ρD*_*0*_^3^/8*γ*)^1/2^. (**d**) Droplet impact on a rigid superhydrophobic surface at higher impact speeds (*v* = 1.58 m/s), showing breakup and splashing. (**e**) Droplet impact on an elastic superhydrophobic surface at higher impact speeds (*v* = 1.57 m/s), showing substrate oscillation, and early lift-off of the droplet in a pancake shape at reduced contact times (*t*_*c*_ <*t*_*c,th*_ = 6.3 ms).

We first considered droplet impact on stiff and elastic nanostructured superhydrophobic surfaces. The elastic and rigid surfaces were created by coating polymer sheets of thickness *h*_*s*_ = 10–500 μm and a glass slide, respectively, with a commercial nanoparticle spray to render them superhydrophobic, resulting in apparent advancing/receding contact angles of 164 ± 4°/159 ± 3° (see *Methods* for details). Prior to droplet impact testing, the superhydrophobic coating was characterized for its morphology and wetting characteristics. Figure 1a,b show optical and scanning electron microscopy (SEM) images of the superhydrophobic surfaces. Individual nanoparticles with diameters *d* ~30 nm (Fig. 1b) formed macroscopic clusters (Fig. 1a), leading to random, hierarchical surface structures with an overall roughness of *r* ≈ 7.5, where *r* is the ratio of the total surface area to the projected area, and an effective solid-liquid fraction of *f* ≈ 0.05 (for more information on surface roughness, see Supplementary Information, section S.3). Droplet impact on the elastic surfaces differed greatly from impact on the rigid superhydrophobic surface.

Figure 1c shows a droplet with *D*_*0*_ = 1.48 mm impacting the rigid superhydrophobic sample at a speed of *v* = 0.68 m/s, corresponding to *We* = 9.6 and *Oh* = 0.003 ≪ 1 (
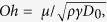
 where *μ* is the droplet dynamic viscosity). As expected, impact causes the droplet to spread laterally, undergo kinetic-to-surface energy conversion due to additional surface area creation, and reach a maximum diameter *D*_*max*_ at the spreading time *t* = *t*_*spr*_. The droplet then undergoes the reverse process and retracts due to surface-to-kinetic energy conversion and finally lifts off the surface at the theoretical contact time *t*_*c*_ = *t*_*c,th*_ = 2.6 (*ρD*_*0*_^3^/8*γ*)^1/2 ^10,11,12,13. At higher impact speeds, as shown in Fig. 1d with a droplet with *D*_*0*_ = 1.49 mm and *v* = 1.58 m/s (*We* = 51.7), the droplet splashes, *i.e.* breaks up into a core droplet and several satellite droplets after reaching its maximum spreading diameter. The remaining core of the droplet detaches from the substrate at *t*_*c*_ = *t*_*c,th*_. Interestingly, when the droplet impacts the elastic surface, as shown in Fig. 1e with a droplet with *D*_*0*_ = 1.50 mm and *v* = 1.57 m/s (*We* = 51.2), splashing is eliminated by the droplet edge detachment from the surface. Before the droplet can fully retract, the entire droplet has lifted off the surface in a spread (or pancake) shape at a contact time 21% shorter than the theoretical contact time on a rigid superhydrophobic surface.

### Energy Conversion During Impact

Inspired by this unique observation, we hypothesize that substrate elasticity enables a new energy conversion mechanism to come into play during droplet impact. On the elastic surface, two distinct post-impact energy conversion mechanisms exist: kinetic-to-surface within the droplet and kinetic-to-elastic between the droplet and the elastic substrate. If tailored correctly, the elasticity of the substrate can be designed such that the two fundamental energy conversion mechanisms have disparate timescales, allowing for faster vertical momentum transfer from the elastic-to-kinetic energy conversion than from the classical surface-to-kinetic mechanism within the droplet, and thus early droplet lift-off from the surface.

**Figure 2:**
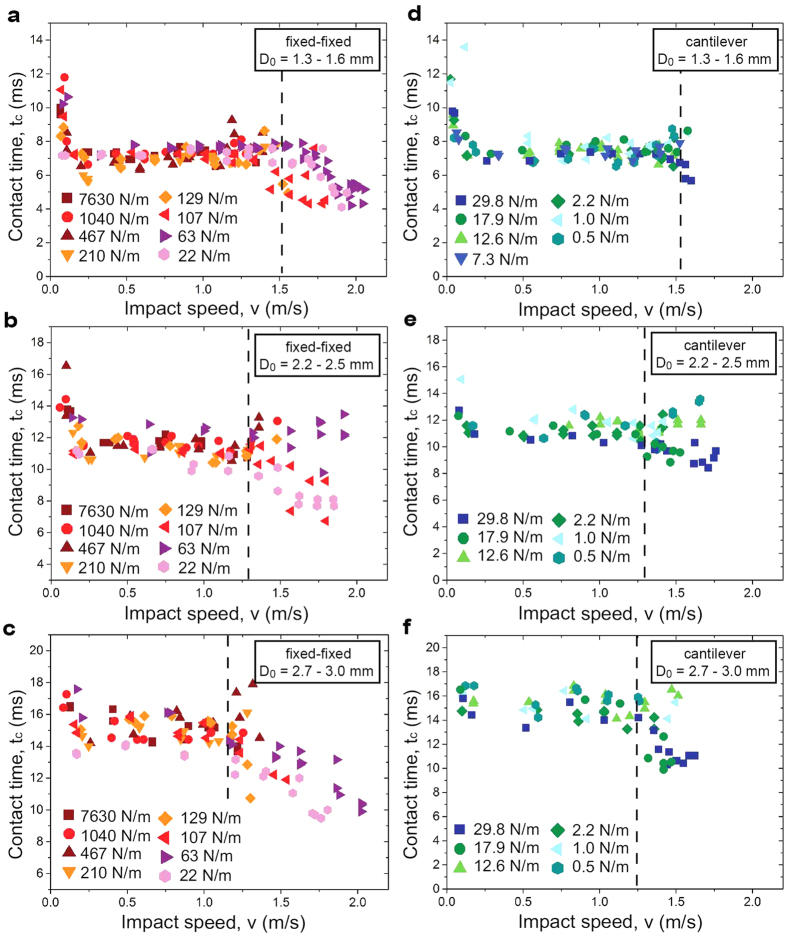
Contact times for impacting droplets for fixed-fixed (**a**–**c**) and cantilever (**d**–**f**) style mounted substrates as a function of impact speed and substrate stiffness for the three droplet diameter ranges. The critical impact speed, *v*_*c*_ (also see Supplementary Information, section S.5), is marked with a dashed line. For impact speeds, *v* >*v*_*c*_, the contact time decreased as a result of the ‘springboard’ effect where the droplet lifted off the surface prior to fully retracting. Measurements were terminated once splashing occurred. Error bars are smaller than the symbol sizes and are not shown. In (**b**), droplets impacting the substrate with stiffness *k* = 63 N/m did so off-center from the axis, inducing a torsion of the substrate rather than an oscillation, and eliminating the possibility for contact time reduction.

In order to systematically study the effect of substrate elasticity on contact times, we conducted droplet impact experiments on elastic surfaces with varying stiffness (0.5< *k* <7630 N/m), fixture mode (fixed-fixed vs. cantilever), as well as varying the droplet size (1.3< *D*_*0*_ <3.0 mm) and impact speed (0.05< *v* <2.1 m/s), corresponding to 0.05< *We* <115. For all experiments, the impacting droplet Ohnesorge number *Oh* ≪ 1, such that viscous forces were negligible when compared to capillary or inertial forces. Figure 2 summarizes the contact times for droplet impact on fixed-fixed substrates (a, b, c) and cantilever-style substrates (d, e, f). For substrate stiffness 20< *k* <150 N/m and impact speeds greater than a critical impact speed, *v*_*c*_, contact times were reduced when compared to impact on a rigid superhydrophobic surface (*k* = 7630 N/m). For low impact speeds (*v* <0.2 m/s), contact times rapidly decreased with increasing speeds. Droplets in this speed regime behaved similarly to an elastic ball and did not spread (see Supplementary Video S1). For 0.2< *v* <*v*_*c*_, substrate mounting and stiffness had no effect on the droplet contact time (see Supplementary Video S2 and Video S3), which was in excellent agreement with the inertial-capillary scaled contact time, *t*_*c*_ = *t*_*c,th*_. For *v* > *v*_*c*_, splashing occurred for substrates having *k* <20 N/m and *k* >150 N/m (see Supplementary Video S4), while on substrates having moderate stiffness (20< *k* <150 N/m), splashing was delayed and contact times decreased linearly with increasing impact speeds (see Supplementary Video S5). The experiments were terminated once splashing occurred. Our data shows that droplet contact times can be halved when compared to droplet impact at lower impact speeds and on rigid superhydrophobic substrates. The reduction in contact time was observed on both fixed-fixed and cantilever-style substrates and was found to weakly depend on the axial impact location of the droplet due to an increase in substrate stiffness with decreasing distance to the mount (see Supplementary Information, section S.4). It is important to note that the stiffness of the substrate did not directly influence the slope and magnitude of the decrease in contact times. Furthermore, droplet spreading times were not affected by the substrate elasticity and remained constant for all substrate mounting and elasticities, in agreement with previous studies (see Supplementary Information, section S.5)^1^,^17^.

In addition to contact time reduction, Fig. 2 shows that splashing, i.e. droplet breakup and creation of satellite droplets, occurs at higher impact speeds on elastic substrates than on rigid substrates, which has been reported previously for ethanol droplets impacting a circular membrane^18^. In the present study, the increase in the splashing threshold speed on fixed-fixed substrates was observed for substrate stiffness up to 130 N/m. The elasticity of the substrate enables kinetic-to-elastic energy conversion between the droplet and the substrate at early stages of impact. Hence, not as much energy is left over for kinetic-to-surface energy conversion, resulting in a decreased spreading inertia and increased critical splashing speed^18^. For all droplet sizes, splashing initiated at higher impact speeds on fixed-fixed when compared to the cantilever-style substrates due to more efficient kinetic-to-elastic energy conversion on the former mounting configuration.

### Mechanism of Elasticity Mediated Contact Time Reduction

In order to better understand the physical mechanism of contact time reduction, we measured the dynamics of the elastic substrate during impact and developed a simple oscillator model of substrate motion. For the fixed-fixed and the cantilever substrates, the eigenfrequencies *f*_*0*_ can be computed as 
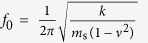
and 
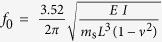
, respectively, where *m*_*s*_, *L*, *ν*, *E*, and *I* are the mass, length, Poisson’s ratio (*ν*_*PMMA*_ = 0.38), Young’s modulus (*E*_*PMMA*_ = 3 GPa), and area moment of inertia (*I* = *wh*_*s*_^3^/12) of the substrate, where *w* is the width and *h*_*s*_ the thickness of the substrate. Table 1 lists the calculated and observed eigenfrequencies of the substrates, which are in excellent agreement, giving us confidence in the validity of the harmonic oscillator approximation.

**Figure 3:**
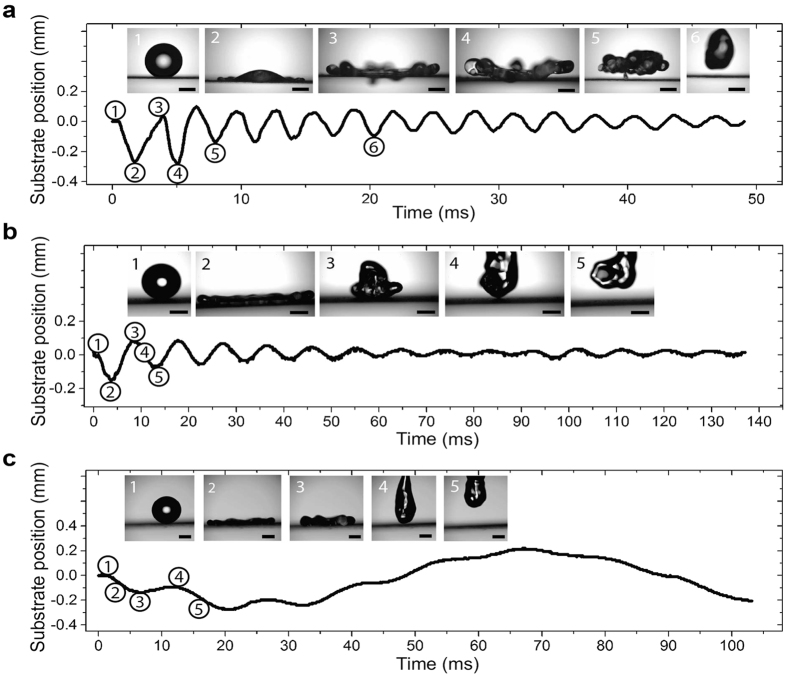
Substrate response and droplet shapes after droplet impact (scale bar 1 mm for all inset images). (**a**) Fixed-fixed substrate with *k* = 107 N/m, *D*_*0*_ = 2.2 mm and *f*_*0*_ = 325 Hz. Inset: Time lapse images of the droplet impact. The substrate starts a harmonic oscillation at its natural frequency shortly after droplet impact. The droplet spreads, and lifts off the surface in a pancake shape (*springboard effect*). (**b**) Cantilever substrate with *k* = 29.8 N/m and *D*_*0*_ = 2.1 mm. The substrate oscillates at its natural frequency, *f*_*0*_ = 112 Hz. Inset: Time lapse images of the droplet impact showing that droplet lift-off does not occur in a pancake shape, but is distinct from the retraction behavior on a rigid substrate. (**c**) Cantilever substrate with *k* = 2.2 N/m and *D*_*0*_ = 2.4 mm. The substrate is so elastic that the droplet impact activates substrate oscillations with both the natural (*f*_*0*_ = 11 Hz) and higher order frequencies (*f*_*1*_ = 68 Hz). Even more elastic substrates exhibit even higher order modes, *f*_*2*_ = 178 Hz (not shown). Inset: Time lapse images of the droplet impact showing spreading, retraction, and lift-off behavior similar to that on rigid superhydrophobic surfaces.

Figure 3 shows the substrate response for three different substrate and impact conditions. Upon impact, the droplet exerts a force on the substrate, which causes the substrate to oscillate. The upward motion of the substrate during oscillation governs the contact time reduction of an impacting droplet, acting to accelerate the flattened droplet (~*D*_*max*_) in the vertical direction against gravity. The added upward force from the elastic substrate causes the droplet to detach before fully undergoing surface-to-kinetic energy conversion. During early departure, the droplet remains in a spread, pancake-like shape (see Supplementary Videos S5). We refer to this early lift-off mechanism as the *springboard effect*, or *springboarding*, taking inspiration from a springboard where the vertical acceleration of an elastic membrane or spring (elastic-to-kinetic energy exchange) helps a jumper to rebound. It is important to note that although analogous droplet springboarding, or trampolining has been observed on suspended soap films^19^, the springboard effect shown here differs fundamentally from evaporation induced droplet trampolining^20^. The droplet images in Fig. 3a illustrate the coupling between the oscillation of an elastic substrate in the fixed-fixed configuration (*k* = 107 N/m) and droplet lift-off in the pancake-shape. After reaching the maximum spreading diameter (3), the edges of the droplet detach (4) near a minimum substrate position. The subsequent upward motion of the substrate supports the detachment of the center of the droplet and results in early droplet lift-off (5). Figure 3b,c show the cantilever-style substrate responses for *k* = 29.8 N/m and *k* = 2.2 N/m, respectively. While for the high stiffness cantilever (*k* = 29.8 N/m), droplet lift off did not occur in the pancake shape, yet contact time reduction was observed. However, droplet impact on the low stiffness substrate (*k* = 2.2 N/m) did not have a reduced contact time. Furthermore, while the stiffer cantilever oscillated at its natural eigenfrequency (*f*_*0*_ = 112 Hz), the softer substrate showed an additional higher order oscillation (*f*_*0*_ = 11 and *f*_*1*_ = 68 Hz). The time scales of droplet spreading and recoil (~10 ms) were much shorter than the first order oscillation timescale (~100 ms) for the low stiffness substrate, resulting in the inability to accelerate the droplet upwards (see Supplementary Video S6), and enabling full droplet recoil before lift-off with similar contact times as those on a rigid superhydrophobic surface^21^,^22^. To enable contact time reduction, our results show that the substrate oscillation and droplet impact timescales must be on the same order of magnitude.

An additional mechanism for contact time reduction can be found by studying the droplet dynamics at high impact speeds. We first determined the critical speed for the onset of contact time reduction, *v*_*c*_, which was a function of the initial droplet diameter (Fig. 2). As shown in Figs 1e and 3a, and explained above, early lift off initiates at the edge of the droplet, analogous to the initial phase of droplet breakup during splashing. When splashing occurs, viscous drag decelerates the rim of the spreading droplet while the edges deform upwards and away from the surface. Although surface tension forces act to minimize the liquid/air surface area, inertia of the spreading droplet coupled with Kelvin-Helmholtz instabilities on the droplet periphery enable the breakup and the formation of satellite droplets, *i.e.* splashing^23^,^24^,^25^. For splashing to occur, the splash parameter, *K* = *WeRe*^1/2^, where the droplet Reynolds number is *Re* = *ρD*_*0*_*v*/*μ*, must be greater than a critical parameter *K*_*c*_. The critical parameter depends on the substrate roughness, and can be approximated as, *K*_*c*_ ≈ 3,600 for the substrates used in this study^26^,^27^,^28^. During droplet bouncing in the present experiments, droplets initiate their lift-off at the edges of the droplet at maximum spreading, as can be seen in Figs 1e and 3a; a behavior similar to the first step during splashing (also see Supplementary Video S5). During spreading, the leading edge of the radial liquid flows over a small layer of air beneath it which provides lift to the advancing contact line^29^. The lift-force created by the spreading rim acts against gravity and facilitates the subsequent substrate oscillation-driven lift-off of the entire droplet. We can thus link the critical splashing parameter to the onset of contact time reduction to obtain *v*_*c*_ = 2.1/*D*_*0*_^0.6^, where *D*_*0*_ is the droplet diameter in units of millimeters, and *v*_*c*_ is the critical droplet impact speed in units of meters per second. For a detailed derivation of the critical impact speed, including a dimensional analysis, and comparison to experimental data, see Supplementary Information, section S.6. The scaling *v*_*c*_ ~ *D*_*0*_^*−*0.6^ suggests that, for very small droplets, a reduction in contact time might not be possible due to the high critical speed required to overcome the dominant surface tension forces at small length scales. Indeed, it has been observed that micrometric droplets do not splash, even at impact speeds exceeding 10 m/s^30^. Droplet sizes in many applications, including spray cooling, range from 50 to 200 μm with typical impact speeds of 5 to 25 m/s, exceeding the critical impact speed required for contact time reduction^31^,^32^. Due to scale invariance of droplet impact phenomena for droplets bigger than approximately 50 μm^30^, we expect springboarding for droplet size ranges often found in spray cooling. For smaller droplets (*D*_*0*_ <50 μm), viscous forces begin to dominate droplet inertia and surface tension, resulting in potential elimination of droplet springboarding^33^.

## Discussion

**Figure 4:**
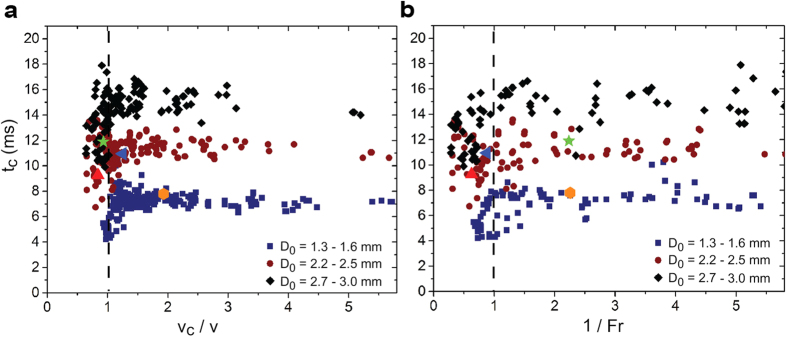
Droplet impact conditions for contact time reduction. (**a**) Contact times for the droplet size regimes as a function of the ratio of critical speed to impact speed, *v*_*c*_/*v*. For the springboard effect to occur, the condition *v*_*c*_/*v* ≤ 1 must be met. (**b**) Contact times for the three droplet size regimes as a function of the inverse Froude number. To reduce contact times, 1/*Fr* ≤ 1 must be met. (**a**,**b**) also include four selected points that represent experimental conditions where: both dimensionless parameters (*v*_*c*_/*v* and 1/*Fr*) were met (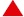), only the first (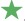) or second (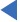) condition were met, and where neither of the conditions were met (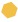). The experimental data points include all experimental runs, *i.e.* fixed-fixed and cantilever for all substrate stiffnesses. Error bars in the experimental data points are smaller than the symbol size, and are not shown.

In an effort to explain the physics governing the springboard effect on elastic substrates, we identified two fundamental conditions needing fulfilment to enable contact time reduction. The first condition relates the droplet impact speed to the critical impact speed while the second condition relates the droplet and substrate inertia to the gravitational body force. Figure 4a shows the experimentally measured contact time as a function of the impact speed normalized by the critical speed. To achieve contact time reduction, the impact speed must exceed the critical speed:


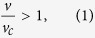


where *v*_*c*_ = 2.1/*D*_*0*_^0.6^. For droplets having diameters of *D*_*0*_ ≈ 1.5 mm and *D*_*0*_ ≈ 2.8mm, Eq. (1) can be described by *We* >40 and *We* >60, respectively. The need to exceed the critical velocity in order to achieve droplet springboarding does not directly depend on the substrate characteristics, and stems from a balance of kinetic and surface energy required to suppress splashing. If droplet impact occurs at an oblique angle from the surface-normal vector, spreading and splashing of droplets remains radially symmetric due to low droplet-surface adhesion^34^. The surface-normal component of impact speed must be used in the critical velocity criterion (*v* cos*^2^(α)*/*v*_c_ ≥ 1), where *α* is the tilt angle of the substrate to the horizontal, to ensure contact time reduction of oblique droplet impact (see Supplementary Information, section S.7).

The second condition (Fig. 4b) requiring fulfilment for contact time reduction depends on the substrate stiffness and oscillation frequency after impact. As described earlier, the vertical momentum transfer from the substrate to the droplet causes the droplet to lift-off in the pancake shape, leading to the reduction in contact time. We can quantify the vertical momentum transfer by defining an experimental Froude number (*Fr*) as the ratio of substrate inertia to the gravitational body force acting on the droplet during impact. The substrate inertia is defined as the vertical momentum transfer from the oscillating substrate to the droplet in the upward direction against gravity. For droplets to lift off with lower contact times, i.e. in a pancake shape, the upward substrate inertia must exceed the downward gravitational body force, thus the second condition can be written as:


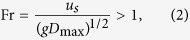


where *u*_*s*_ = 2π*f*_*0*_*δ*_*max*_ is the maximum substrate velocity during oscillation. The maximum impact force of the droplet on the substrate, *F*_*0*_ = π*ρ(D*_*0*_*/2)*^2^*v*^2^, is used to determine the maximum deflection of the substrate, 
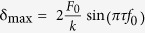
, where *τ* = *D*_*0*_/*v* is the crash time of the droplet^35^,^36^. A comparison of the experimental and calculated maximum substrate deflection showed that the *sin* term can be neglected for fixed-fixed substrate configurations (see Supplementary Information, section S.8). The maximum spreading diameter, *D*_*max*_, is obtained from kinetic and surface energy conservation before impact and at maximum spreading, resulting in the dimensionless spreading parameter *ξ*_*max*_ = *D*_*max*_/*D*_*0*_ ~ *We*^1/2^. Indeed, fitting the theoretical dimensionless spreading parameter to our experimental data (see Supplementary Information, section S.4) yielded *ξ*_*max*_ = 0.22 *We*^1/2^ + 0.7. Equations (1) and (2) enable us to predict whether impacting droplets will enter the springboard regime and consequently have reduced contact times during impact when impacting elastic substrates at rest. The Froude number criterion represents the coupling of time scales of substrate oscillation and droplet contact time as well as the amplitude of substrate oscillation caused by the impacting force of droplets. When the substrate is oscillating prior to droplet impact, caused, for example, by environmental conductions such as wind or vibrations, these external forces would need to be taken into account when determining the contact time reduction criteria.

Figure 4a,b include four select data points where i) both conditions are met (red triangle), ii) only the first condition is met (green star), iii) only the second condition is met (blue triangle), and iv) neither condition is met (orange hexagon). The experimental results show that in order to reduce contact times, both dimensionless conditions have to be satisfied. If designed properly, following the two dimensionless conditions presented above, elastic substrates can lead to a 2-fold reduction in contact time compared to impact on rigid superhydrophobic surfaces. Thus, we envision that the contact time reduction on elastic surfaces not only extends our fundamental understanding of wetting phenomena, but also offers potential for a wide range of applications including anti-icing^4^,^37^,^38^,^39^, self-cleaning^40^, and heat transfer enhancement (see Supplementary Information, section S.9). Although demonstrated here for membrane and cantilever based systems, future studies on elastic solid substrates such as gels or elastomers is needed to verify the contact time reduction criteria on volumetric based elastic materials that would provide reduced sensitivity to impact location and environmental forces on contact time reduction.

## Methods

### Fabrication of Elastic Substrates

The commercially available superhydrophobic coating NeverWet^©^ was sprayed onto different glass and polymer substrates. NeverWet consists of a flat base coat without nanoparticles (water contact angle *θ*_*a*_/*θ*_*r*_ = 105 ± 3°/66 ± 4°) and a top coat consisting of conformally coated hydrophobic nanoparticles with diameters of ≈50 nm. On each sample, 3 base coats and 3–4 top coats were applied, resulting in apparent advancing and receding contact angles of *θ*_*a*_^*app*^/*θ*_*r*_^*app*^ = 164 ± 4°/159 ± 3°. Polymer sheets (GoodFellow) of various thickness were cut into rectangles with dimensions listed in Table 1 and used as substrates. The sheets used in this study were: 500 μm polymethylmethacrylat (PMMA), 250 μm PMMA, 175 μm PMMA, 100 μm polyhydroxybutyrate (PHB), 36 μm PMMA, and 10 μm PMMA. A glass microscope slide coated with NeverWet served as a rigid baseline substrate to ensure that observations on other substrates could be clearly attributed to elasticity, and not the coating itself. The geometric dimensions, including substrate thickness *h*_*s*_, substrate width *w*, substrate length *L*, mass of the substrate *m*_*s*_ (density *ρ*_*PMMA*_ = 1180 kg/m^3^), and distance of impact to the fixture *s* are listed in Table 1.

### Surface Characterization

Scanning electron microscope (SEM) images were taken with a Quanta 450 FEG ESEM in high vacuum mode. The surface roughness on the macroscale (~μm) was characterized with an Alicona Infinite Focus 3D microscope. The lateral resolution with a 50x lens was 1 μm and the vertical resolution was 40 nm. Multiple scans were combined to measure a larger area. The roughness was analyzed with the internal software provided by Alicona. It is important to note that the 3D microscope’s resolution is much larger than the average nanoparticle size, thus, the roughness determined with the Alicona 3D microscope pertains to differences in microstructure only, for example due to nanoparticle clustering, and does not reflect the underlying nanostructure. The nano-roughness was determined using atomic force microscopy (AFM) (Cypher by Asylum Research) in tapping mode at a scan rate of 0.5 Hz, a scan speed of 5 μm/s, and a step size of 15.6 nm. The roughness was analyzed with the internal software provided by Asylum Research.

### Characterization of Substrate Stiffness

Droplet impact was studied with two substrate mountings: fixed-fixed along the short edges of the substrate, and fixed-free along one short edge of the substrate, *i.e.* cantilever-style mounting. The stiffness of the cantilever was determined from geometrical considerations, with the area moment of inertia *I* = *wh*_*s*_^3^/12, where *w* and *h*_*s*_ are the width and thickness of the substrate. The stiffness was calculated as *k* = 3*EI*/*s*^3^, where *E*_*PMMA*_ ≈ 3 GPa is the elastic modulus of the substrate and *s* is the distance between the centerline of droplet impact and the fixed end. The stiffness of the fixed-fixed substrate depended on the substrate geometry and mounting tension, and was thus experimentally measured. A cylindrical hook with a diameter of 3.3 mm, connected to a force gauge (MG025, Mark-10), contacted the substrate from below, at the location of droplet impact. The hook-gauge-assembly had a stiffness of *k*_*F*_ ≈ 4000 N/m. The force gauge was mounted on a linear translation stage (Thor Labs) and was slowly moved upwards to a total displacement, Δ*d*_*tot*_, at which point the total force *F* was recorded. The stiffness of the fixed-fixed substrate was determined to be *k* = *F* / (Δ*d*_*tot*_ – *F*/*k*_*F*_). Table 1 lists the calculated and experimentally measured stiffness for each substrate. A schematic of the setup is shown in Supplementary Information, section S.2.

### Droplet Impact Measurements and Image Analysis

Three needles having 33 (G33), 25 (G25) and 20 (G20) gauge with outer diameters of 210 μm, 515 μm and 908 μm, respectively, were connected to a syringe pump at a flow rate of 50 μL/min (Pico Plus, Harvard Apparatus). Individual droplets with diameters ranging from 1.3 to 3 mm (see Table 1) were formed at the tip of the needles and detached due to gravitational force. The height of the needle above the surface was varied between 3 and ~300 mm, resulting in impact speeds ranging from 0.05 to 2.1 m/s. Only data for non-splashing droplets was used in this study. The droplets impacted the stationary substrate in the center between the mountings in the fixed-fixed case and at a specified distance *s* from the single-sided mounting for the cantilever case. A high speed camera (Phantom v711, Vision Research) coupled to a 1–5x tele lens (Canon) recorded the impacting droplets at a frame rate of 9500 fps, resolution of 1024 × 768 pixels, and exposure time of 30 μs. The images were calibrated for each experiment with respect to the outer diameter of the dispensing needle, obtaining a resolution ranging from 6 to 10 μm/pixel. The images were analyzed with a Matlab code to determine the initial diameter, impact speed, maximum spreading, and substrate deflection, while manual analysis was required to obtain the spreading and contact times.

## Additional Information

**How to cite this article**: Weisensee, P. B. *et al.* Water droplet impact on elastic superhydrophobic surfaces. *Sci. Rep.* **6**, 30328; doi: 10.1038/srep30328 (2016).

## Supplementary Material

Supplementary Information

Supplementary Video S1

Supplementary Video S2

Supplementary Video S3

Supplementary Video S4

Supplementary Video S5

Supplementary Video S6

Supplementary Video S7

Supplementary Video S8

## Figures and Tables

**Table 1:** Substrate and droplet parameters: fixture mode (f-f: fixed-fixed, c: cantilever), substrate geometries, and average droplet sizes for each experimental set.

Fix	*h*_*s*_(μm)	*w*(mm)	*L*(mm)	*m*_*s*_(kg)	*s*(mm)	*k*(N/m)	*f*_*0*_ (Hz)	*f*_*0,obs*_(Hz)	*D*_*0*_(G33) (mm)	*D*_*0*_(G25) (mm)	*D*_*0*_(G20) (mm)
f-f	1100	25	53	3.69e-3	26.5	7630	247	—	1.49	2.37	2.84
f-f	500	27	64	1.08e-3	32.0	1040	169	—	1.46	2.38	2.89
f-f	250	26	59	5.03e-4	29.5	467	166	244	1.40	2.34	2.89
f-f	175	25	65	3.89e-4	32.5	210	126	147	1.45	2.29	2.72
f-f	100	25	71	2.78e-4	35.5	129	117	96	1.52	2.33	2.75
f-f	36	7.5	63	3.57e-5	31.5	107	298	325	1.51	2.23	2.76
f-f	10	15	53	3.56e-5	26.5	63	229	—	1.38	2.39	2.93
f-f	10	20	110	9.81e-5	55.0	22	81	93	1.47	2.00	2.50
c	175	15	20	1.03e-4	15.0	29.8	122	111	1.44	2.15	2.57
c	175	25	20	6.20e-5	15.0	17.9	122	105	1.55	1.96	2.40
c	175	25	65	3.36e-4	20.0	12.6	11	8	1.50	2.38	2.87
c	175	25	65	3.36e-4	24.0	7.3	11	10	1.44	—	—
c	175	25	65	3.36e-4	35.5	2.2	11	11	1.59	2.36	2.74
c	175	25	65	3.36e-4	46.0	1.0	11	11	1.51	2.36	2.97
c	175	25	65	3.36e-4	59.5	0.5	11	10	1.52	2.35	2.71
c	100	6.5	13	1.07e-5	var.	var.	165	140	—	2.3–2.5	—
