# Supplementary material for: Water droplet impact on elastic superhydrophobic surfaces
Source: Sci Rep. 2016 Jul 27;6:30328. doi: 10.1038/srep30328 (PMC4962083; doi:10.1038/srep30328)
Supplement: Supplementary Information [file srep30328-s1.pdf]

Water droplet impact on elastic superhydrophobic surfaces

Supplementary Information

Patricia B. Weisensee[1], Junjiao Tian[1], Nenad Miljkovic[1,2,*] and William P. King[1,*]

[1]*Department of Mechanical Science and Engineering, University of Illinois at Urbana-Champaign*

[2]*International Institute for Carbon Neutral Energy Research (WPI-I2CNER), Kyushu University, 744 Motooka, Nishi-ku, Fukuoka 819-0395, Japan*

[*] wpk@illinois.edu, nmiljkov@illinois.edu

S.1 High Speed Videos

All high speed videos were captured at 9,500 fps at a resolution of 1024 x 768 pixels, and an exposure time of 30 μs. All videos are played back at 30 fps (> 300x slowed down).

Video S1. Droplet impact (D_0 = 2.30 mm, v = 0.13 m/s) on a rigid, superhydrophobic NeverWet coated glass substrate. Slow impact (v < 0.5 m/s) is characterized by quasi-elastic droplet rebound. In this regime, $t_{spr} \approx t_c/2$, where the spreading time is defined as the time when the contact area between the droplet and substrate is maximum. In the quasi-elastic regime, differences in impact dynamics between rigid and elastic substrates are negligible.

Video S2. Droplet impact (D_0 = 2.31 mm, v = 0.84 m/s) on an elastic, superhydrophobic NeverWet coated 175 μm PMMA substrate in the fixed-fixed mounting condition with a stiffness k = 210 N/m. Droplet impact at moderate velocities (0.5 < v < 1.5 m/s) results in the typical spreading and retraction behavior with liquid jetting during recoil. For moderate impact velocities, $t_{spr} \approx t_c/4$, and differences in impact dynamics between the rigid and elastic substrates are negligible.

Video S3. Droplet impact (D_0 = 2.37 mm, v = 1.62 m/s) on an elastic, superhydrophobic NeverWet coated 175 μm PMMA substrate in the cantilever mounting condition with a stiffness k = 29.8 N/m. At high droplet impact speeds (v > 1.5 m/s), just before splashing and droplet breakup occurs, spreading times slightly increase due to partial energy transfer from the droplets to the substrate (initiating oscillation) and a reduction in the droplet spreading kinetic energy.

Video S4. Droplet impact (D_0 = 2.40 mm, v = 1.76 m/s) on an elastic, superhydrophobic NeverWet coated 175 μm PMMA substrate in the cantilever mounting condition with a stiffness k = 29.8 N/m. At high velocities, inertial forces during spreading overcome capillary forces, and the droplet breaks up into many smaller satellite droplets.

Video S5. Droplet impact (D_0 = 1.98 mm, v = 1.62 m/s) on an elastic, superhydrophobic NeverWet coated 10 μm PMMA substrate in the fixed-fixed mounting condition with a stiffness k = 22 N/m. At high droplet impact speeds ($v > v_c$), and before splashing occurs, droplets lift off in a pancake shape before fully retracting. The substrate oscillation generates an upward force on the droplet, allowing it to skip the capillary-to-inertial energy conversion step and depart early. We observe up to 2-fold decreases in contact time compared to droplet impact at lower speeds or on rigid superhydrophobic substrates. We call this mechanism of early lift-off the *springboard effect*, taking inspiration from a springboard where the vertical acceleration of the elastic spring or membrane (elastic-to-inertial energy exchange) helps the athlete to rebound.

Video S6. Droplet impact (D_0 = 1.54 mm, v = 1.47 m/s) on an elastic, superhydrophobic NeverWet coated 175 μm PMMA substrate in the cantilever mounting condition with a stiffness k = 0.5 N/m. The very low substrate stiffness allows the impacting droplet to excite both the first and second order substrate oscillation modes. The time scales of droplet spreading and recoil (~ 10 ms) are much shorter than the first order oscillation (~ 100 ms), resulting in the inability to accelerate the droplet upwards and reduce contact time.

Video S7. Droplet impact (D_0 = 2.53 mm, v = 2.19 m/s) on an elastic, superhydrophobic NeverWet coated 100 μm PMMA substrate in the cantilever mounting with an eigenfrequency of f_0 = 185 Hz, and at an angle of 14.9° to the horizontal. Despite the substrate inclination the springboard effect with reduction in contact time can be clearly seen as the droplet lifts off the surface before fully retracting.

Video S8. Droplet impact (D_0 = 2.52 mm, v = 2.09 m/s) on an elastic, superhydrophobic NeverWet coated 100 μm PMMA substrate in the cantilever mounting with an eigenfrequency of f_0 = 185 Hz, and at an angle of 25.5° to the horizontal. Due to the inclination and low friction of the substrate the droplet slides along the substrate after impact and prior to lift-off. Despite the substrate inclination the springboard effect with reduction in contact time can be clearly seen as the droplet lifts off the surface before fully retracting.

S.2 Experimental Setup

Figure S1 illustrates the measurement setup and substrate dimensions for the droplet impact experiments. The setup is described in detail in the Methods section in the manuscript and the geometric substrate dimensions are listed in Table 1.

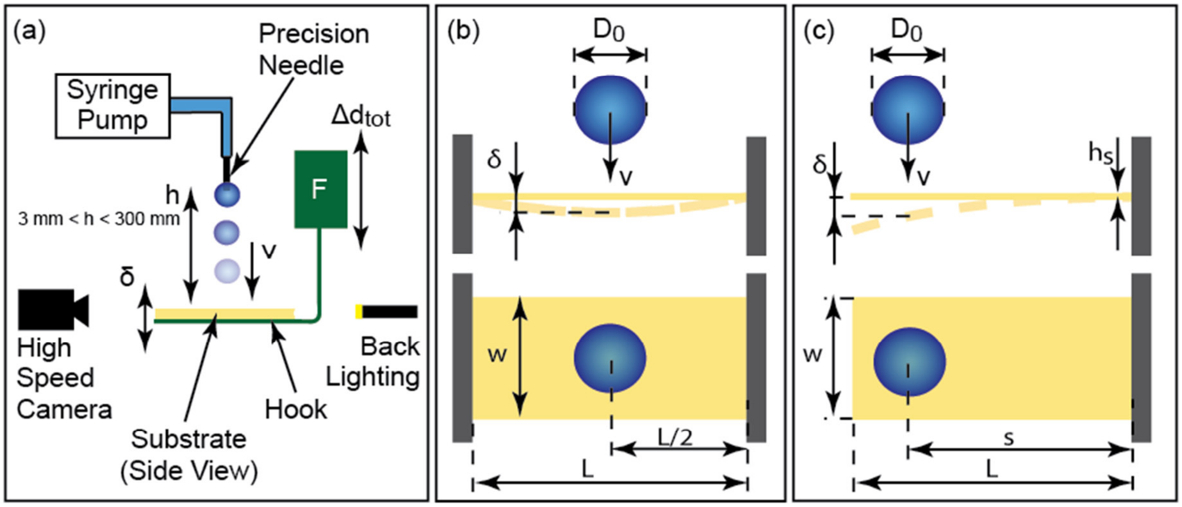

Figure S1. **Experimental setup and substrate geometries.** (a) Droplets, generated with a syringe pump, fall unto the substrate from varying heights (3 mm < h < 300 mm) with the impact speed v (0.05 < v < 2 m/s). A high speed camera records the impact and the deflection of the substrate, δ. To measure the stiffness of a fixed-fixed substrate, a hook is inserted from below and a force gauge (force F) is displaced by means of a linear translational stage (Δd_{tot}). (b) and (c) Geometric dimensions of fixed-fixed (membrane) and fixed-free (cantilever) substrates. Substrates are clamped with a uniform pressure distribution over the width of the substrate. The deflection of the substrates is measured at the location of impact.

S.3 Surface Characterization

Figure S2 shows three dimensional (3D) shaded surface plots of a glass microscope slide coated with the superhydrophobic NeverWet coating. Figures S2a and b reveal nanoparticle clustering with up to 60 µm tall features, randomly distributed over the surface, leading to a macroscopic roughness of $r_{\mathrm{macro}} = 1.2$, defined as the ratio of the total macroscopic area to the

projected area. At the microscale, individual nanoparticles with diameters d ~ 30 nm form smaller clusters with re-entrant structures that have a characteristic roughness of $r_{\mathrm{micro}} = 1.5$ (Fig. S2c and d). At the nanoscale, *i.e.* the individual nanoparticle level, we can estimate the roughness by modeling the nanoparticles as a monolayer of spheres in contact with each other. With the surface area of an individual nanoparticle, πd^2, and a projected area d^2, the characteristic roughness is $r_{\mathrm{nano}} = (\pi d^2 + d^2)/d^2 = 4.14$. The hierarchical nature of the surface provides an effective total roughness of $r = r_{\mathrm{macro}} \cdot r_{\mathrm{micro}} \cdot r_{nano} \approx 7.5$. Using the values of the advancing contact angles on a rough (θ_a^{app} = 164°) and a smooth (θ_{a} = 105°) surface, we estimate the effective solid fraction of the superhydrophobic surface to be $f = (cos\theta_a^{app} + 1)/(cos\theta_a + 1) \approx 0.05$. On the hierarchical superhydrophobic structures, the droplet resides in the non-wetting Cassie-Baxter state (CB), where air pockets beneath the droplet prevent wetting and are responsible for reduced contact line pinning during droplet impact, resulting in efficient capillary-to-inertial energy transfer during droplet recoil. In the case of the Wenzel-state (W), the droplet would wet the surface structures and pin to the substrate. The observed Cassie type wetting behavior is consistent with the calculated preferred wetting state $E^* = cos\theta_a^{CB}/cos\theta_a^{W} = cos\theta_a^{app}/(r\cos\theta_{\mathrm{a}}) \approx 0.5 < 1$, where E^* is a dimensionless energy criterion to determine the wetting behavior of structured surfaces[1,2].

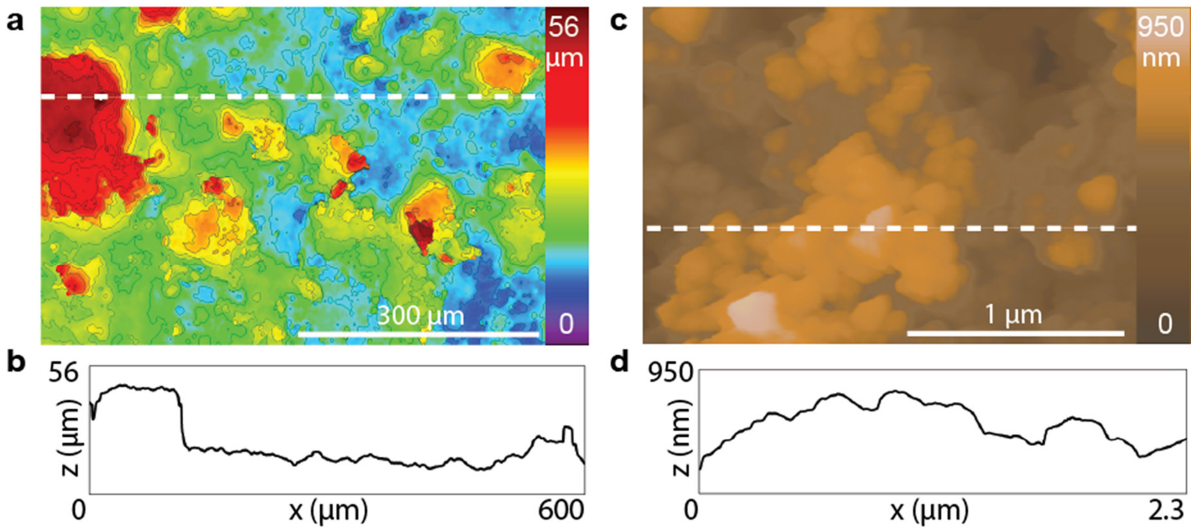

Figure S2. **Surface characterization of the NeverWet superhydrophobic coating.** (a) 3D microscope image showing a typical height distribution and macroscopic roughness. The dashed line indicates the location of the line scan in (b). (c) AFM image showing the microscopic roughness. The dashed line indicates the location of the line scan in (d). Note the different height and length scales in (a) and (c).

S.4 Influence of Axial Impact Location on Contact Times

We expect only minimal effect of the axial impact location on contact times as long as the two criteria for contact time reduction are fulfilled. Impact testing revealed that contact times are nearly constant at $t_c/t_{c,th} \approx 0.6$ for a wide range of impact locations, as can be seen in Fig. S3. The axial location between impact and mount, s, was limited for these experiments by the length of the cantilever and the maximum spreading diameter of the impacting droplet such that the droplet neither falls off the end of the substrate nor makes contact with the mount during spreading. Although not experimentally obtainable here, decreasing s further will result in increased cantilever stiffness at the location of impact, and a corresponding decreased in the droplet Froude number during spreading. For $Fr < 1$ we do not expect to see springboarding and a reduction in contact time.

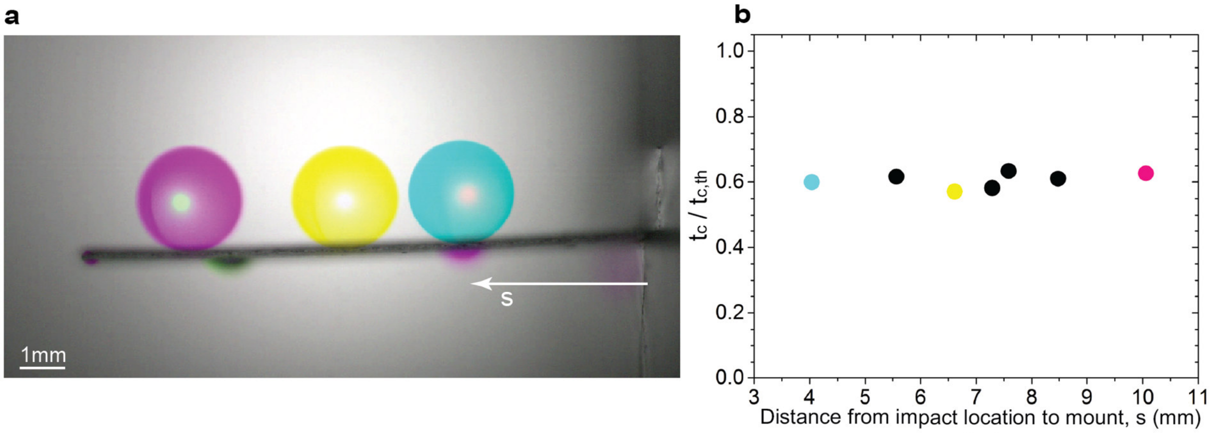

Figure S3. **Effect of Axial Impact Location on Contact Time.** (a) False-color images of three varying droplet impact locations for droplets with $D_0 \approx 2.3$ mm and $v \approx 1.7$ m/s impacting a cantilever-style substrate (h_s = 100 µm, L = 13 mm, w = 6.5 mm, and f_0 = 140 Hz) at an impact location s from the mount. (b) Ratio of contact time over theoretical contact time as a function of the distance between impact location and mount. The range of s was limited by the length of the cantilever and the maximum spreading diameter of the droplets ($D_{max} \approx 6.6$ mm). Since for all impact locations the two criteria for contact time reduction were fulfilled ($v/v_c \approx 1.37$, Fr_{min} = 1.05 for s = 4.0 mm), the droplets were in the springboarding regime with $t_c/t_{c,th} \approx 0.6$.

S.5 Spreading Time and Maximum Spreading Diameter of Droplets

Figure S4a shows the spreading time, t_{spr}, defined as the time taken from initial droplet contact with the surface to the time when the droplet reaches its maximum deformed radius, and average spreading speed for droplets of the three needle sizes. Substrate elasticity had no effect on the spreading time and spreading speed of droplets. At low impact speeds (v < 0.5 m/s, see Video S1), spreading times quickly decreased with increasing impact speed and remained constant for v > 0.5 m/s (see Video S2 and Video S3). At high impact speeds (1.5 m/s < v < v_{splash}) (for splashing, see Videos S4), spreading times slightly increased due to partial energy transfer from the droplets to the substrate (initiating oscillation) and a reduction in the droplet spreading kinetic energy. The constant spreading time at moderate impact speeds can be understood as a balance of impact inertia and capillary forces during spreading. At low impact

speeds (v < 0.5 m/s), gravity plays a dominant role over droplet inertia, making inertial-capillary scaling invalid, and increasing the spreading time[3]. Spreading times were independent of the substrate stiffness and increased with increasing droplet size. The data for the average spreading speed of the droplets (Fig. S4b), defined as the maximum spreading diameter, D_{max}, divided by the spreading time, collapses onto a single curve for all droplet sizes, described by:

$$\frac{D_{max}}{t_{\text{spr}}} = 1.5\ v^{3/4} \tag{S1}$$

Shortly after impact, instantaneous radial spreading speeds are up to 5 times higher than the impact speed, decrease as the droplet rim expands, and become negligible near the point of maximum spreading[4–6]. The average spreading speed $D_{max}/t_{\text{spr}} \sim v^{3/4}$ is obtained from droplet geometry, momentum, and mass conservation during the impact process[7], and does not substantially depend on the elasticity of the substrate[8]. However, the interplay between substrate elasticity, substrate oscillation and droplet liftoff may affect the total contact time, which was characterized next.

The maximum spreading of a droplet during impact can be estimated from an energy balance just before impact and at maximum spreading. Just before impact, at time t = 0, the energy E_0 consists of kinetic energy and surface energy of the droplet:

$$E_0 = \frac{1}{2} m_d v^2 + A_0 \gamma = \frac{\pi}{12} \rho D_0^3 v^2 + \pi D_0^2 \gamma. \tag{S2}$$

At maximum spreading, the droplet can be approximated as a cylinder with height h. Approximating that $h \ll D_{max}$, and neglecting the elastic energy stored in the substrate, the energy at maximum droplet spreading is:

$$E_{spr} = A_{spr} \gamma \approx \frac{\pi}{2} D_{max}^2 \gamma. \tag{S3}$$

By applying the conservation of energy, $E_0 = E_{spr}$, and dividing by $\pi D_0^2 \gamma$, it follows that:

$$\frac{\rho D_0 v^2}{12\gamma} + 1 = \frac{1}{2}\left(\frac{D_{max}}{D_0}\right)^2 = \frac{1}{2}\xi_{max}^2. \qquad \text{(S4)}$$

Thus,

$$\xi_{max} = \sqrt{\frac{1}{6}\frac{\rho D_0 v^2}{\gamma} + 2} \sim a\, We^{1/2} + b. \qquad \text{(S5)}$$

Fitting to the data (see Fig. S4c) yields:

$$\xi_{max} = 0.22\, We^{1/2} + 0.7. \qquad \text{(S6)}$$

Figure S4c also includes model results from previous studies on droplet spreading on rigid surfaces. The models by Mao[9] and Ukiwe and Kwok[10] include a Reynolds number dependency, whereas Clanet *et al.*[11] argue that $\xi_{\max} \sim We^{1/4}$, stemming from a modified capillary length that depends on the deceleration of the droplet during spreading as opposed to gravity. In the Weber number range of the current experiments (0.1 < We < 140), both scaling arguments yield similar spreading parameters. However, we find that the $\sim We^{1/2}$ scaling results in better fits for t_{spr} at low impact speeds. We used Eq. (S6) to solve Eq. (S1) and to calculate the spreading time of impacting droplets as:

$$t_{\text{spr}} = 0.67\xi_{\max} D_0 v^{-3/4}\,. \qquad \text{(S7)}$$

Model predictions from Eq. (S7) are included in Fig. S4a with D_0 = 1.5 mm, D_0 = 2.3 mm and D_0 = 2.8 mm. The predicted values and data match well for all impact speeds. Correlations by other researchers either overestimate the spreading time for small impact speeds and/or underestimate t_{spr} at high impact speeds[11,12].

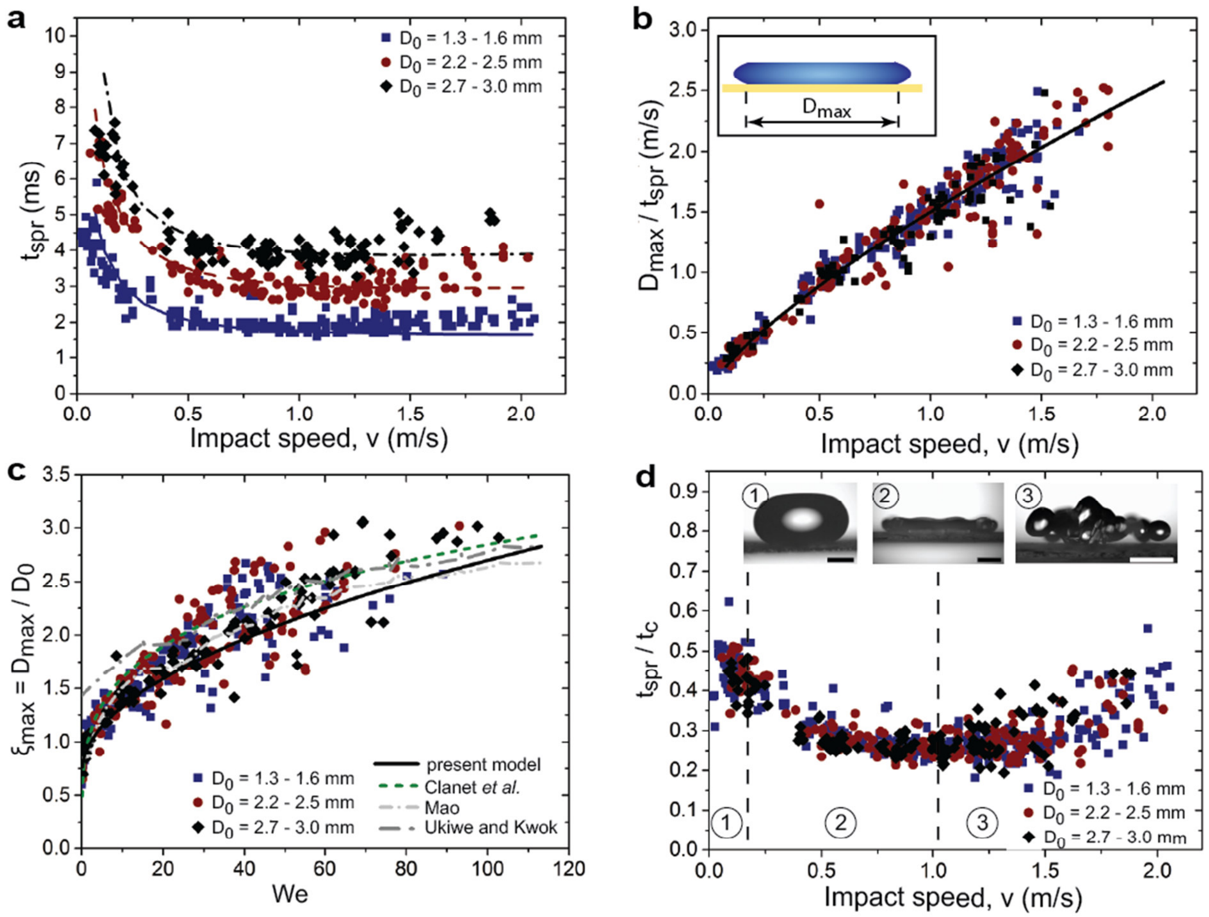

Figure S4. Droplet spreading dynamics. (a) Maximum spreading time, t_{spr}, as a function of impact speed for the three droplet diameter regimes. Eq. (S7) is plotted as lines with D_0 = 1.5 mm (blue), D_0 = 2.3 mm (red) and D_0 = 2.8 mm (black). (b) Normalized maximum spreading parameter as a function of impact Weber number, including the model predictions by Clanet *et al.*[11], Mao[9] and Ukiwe and Kwok[10], as well as the present model fit from Eq. (S6). (c) Average droplet spreading speed, $D_{\max}/t_{\mathrm{spr}}$, as a function of droplet impact speed. All data collapse onto a single curve described by $D_{\max}/t_{\mathrm{spr}} \sim v^{3/4}$ (solid line). Insert: Schematic of the maximum spreading diameter, $D_{\max}$, of the droplet. (d) Ratio of the maximum spreading time to total contact time, $t_{\mathrm{spr}}/t_{\mathrm{c}}$, as a function of We. Inset images show the typical shape of droplets for the three regions 1-3. The scale bar is 1 mm. Data points include all experimental runs, *i.e.* fixed-fixed and cantilever for all substrates stiffnesses. Error bars for the impact speed, spreading time, and maximum spreading diameter are smaller than the symbol sizes and are not included.

Figure S4d shows the ratio of spreading time to total contact time ($t_{\mathrm{spr}}/t_{\mathrm{c}}$). For $We < 10$, i.e. small impact speeds (region 1), $t_{\mathrm{spr}}/t_c \sim 1/2$, due to the symmetric nature of the spreading and recoil phases during elastic droplet rebound (see Video S1). For $We \geq 60$ (region 3), spreading

times remain constant, but contact times decrease, leading to an increase in $t_{\mathrm{spr}}/t_{\mathrm{c}}$ with increasing We. The droplets are in a regime that is characterized by the springboard effect and splashing (see Videos S4 and S5). For $10 \leq We \leq 60$, in the regime of inelastic impact (region 2), the ratio of spreading and contact time are roughly constant (see Video S2). In this region, $t_{\mathrm{spr}}/t_{\mathrm{c}} \sim 1/4$, indicating asymmetry in the impact process and energy dissipative mechanisms that create an imbalance between the inertial-to-capillary energy conversion in the spreading phase, and the capillary-to-inertial energy conversion in the retraction phase.

S.6 Critical Impact Speed for Onset of Springboard Effect

As discussed in the manuscript, the critical impact speed for onset of the springboard effect is similar to the threshold speed for splashing on a rigid surface. For our study, where surface roughness is high, it is reasonable to assume the critical splash parameter K_{c} is close to 3,600.[13] We can then write:

$$K_{\mathrm{c}} = \left(\frac{\rho D_0 v_c^2}{\gamma}\right)\left(\frac{\rho D_0 v_c}{\mu}\right)^{1/2} = 3600\,. \qquad \text{(S8)}$$

For water, ρ = 1000 kg/m^3, γ = 72 mN/m and $\mu = 10^{-3}$ Pa·s, leading to:

$$D_0^{1.25}\, v_c^{2.5} = 2.5 \times 10^{-4}\,. \qquad \text{(S9)}$$

Solving for the critical impact speed, we obtain:

$$v_c = \frac{0.036}{D_0^{0.6}}\,, \qquad \text{(S10)}$$

Converting the droplet diameter from meters to mm, we can write:

$$v_c = \frac{2.27}{D_0^{0.6}}\,. \qquad \text{(S11)}$$

Comparing this to the experimental data reveals that the fit is better for a pre-factor or 2.1 instead of 2.27. Adjusting Eq. (S11) accordingly, we get:

$$v_c = \frac{2.1}{D_0^{0.6}}. \tag{S12}$$

where D_0 is given in units of millimeters, and v_c is in units of meters per second. Figure S5 compares experimental data and model fit for the critical impact speed that is necessary for a reduction in contact times.

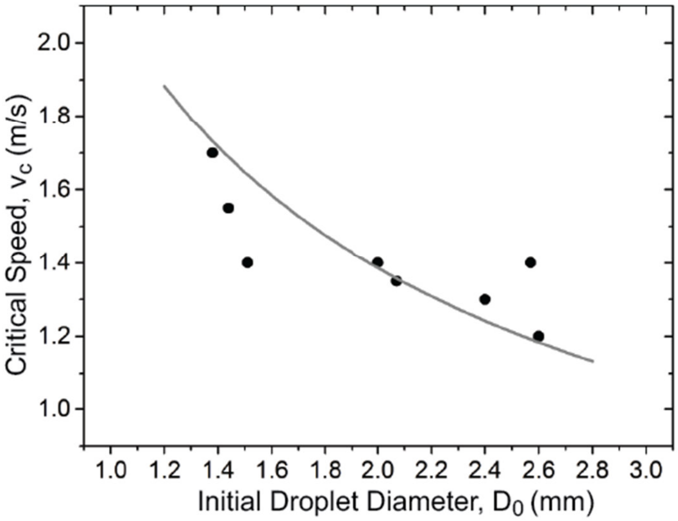

Figure S5. Critical impact speed. A comparison of data and Eq. (S12) for the critical impact speed, v_c, as a function of the initial droplet diameter, D_0. The critical impact speed decreases for increasing droplet diameters as $v_c \sim D_0^{-0.6}$.

S.7 Droplet Impact on Inclined Surfaces

When droplets impact an inclined surface, as shown in Fig. S6a, the first criterion for contact time reduction (Eq. 1 in the manuscript) has to be adjusted for substrate-normal impact speeds. While the normal impact speed scales with $cos(\alpha)$, the critical impact speeds is inversely proportional to $cos(\alpha)$, resulting in a corrected critical velocity criterion:

$$\frac{v\cos^2(\alpha)}{v_c} > 1 \tag{S13}$$

where v_c is described by eq. (S12). Figure S6b compares the ratio of contact time to theoretical contact time for a substrate with two different inclinations (14.9° and 25.5°) using Eq. (1) in the manuscript and the inclination-corrected Eq. (S13), showing that the critical velocity criterion is more accurately fulfilled when using the corrected version. It can also be concluded that, at least for angles $0 < \alpha < 26°$, substrate inclination does not alter the springboarding mechanisms and contact times remain reduced. Videos S7 and S8 show droplet springboarding on inclined substrates with α = 14.9° and 25.5°, respectively.

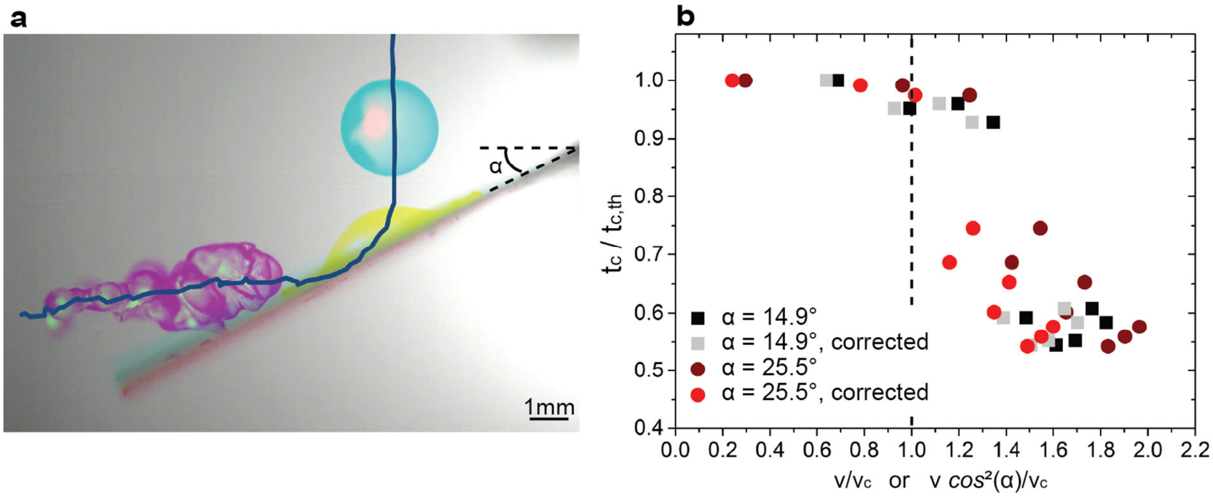

Figure S6. Impact on an inclined surface. (a) Center-of-mass trajectory and three exemplary droplet shapes for a droplet with D_0 = 2.52 mm and v = 2.09 m/s impacting a cantilever-style substrate (h_s = 100 µm, L = 13 mm, w = 6.5 mm, and f_0 = 140 Hz) at an angle of α = 25.5° to the horizontal. After impact, the droplet slides along the substrate before lifting off with a spread shape at $t_c/t_{c,th} \approx 0.6$. (b) Ratio of contact time over theoretical contact time as a function of the critical velocity criterion for two substrate inclinations. For impact on an inclined surface, the critical velocity criterion has to be corrected by a factor of cos²(α) to account for substrate-normal impact velocity for both the impact speed as well as the critical impact speed.

S.8 Maximum Membrane Deflection

Figure S7 compares time-dependent substrate deflections, *i.e.* oscillation, and maximum deflections of selected substrates. Figure S7a shows three examples of the good fit between data and $\delta_{\max} = 2\frac{F_0}{k}\sin(\pi\tau f_0)$ for the maximum substrate deflection when the *sin* term is used only in case of a cantilever-style substrate. Correlations found in literature over- or underpredict the actual deflection for most cases[14,15]. Figures S7b and c show the time dependent substrate deflection and spreading diameter of the droplets during impact. The two substrates have similar stiffness, but different eigenfrequencies, and both show springboard effect behavior for $v > v_c$. For the substrate with the higher natural frequency in Fig. S7b, maximal spreading occurs at the upper peak of the substrate position after the first cycle of oscillation. On the substrate with the lower frequency in Fig. S7c, droplets reach their maximum diameter at the lower dead point of the substrate, after only one quarter of an oscillation cycle. Interestingly, droplets have early lift-off in both cases. The comparison shows that substrate frequency and the springboard effect are not directly coupled. Whether a reduction in contact time occurs or not strongly depends on other factors, such as maximum membrane deflection and impact speed, as represented by the two conditions shown in Fig. 4 and Eqns. (1) and (2) of the manuscript.

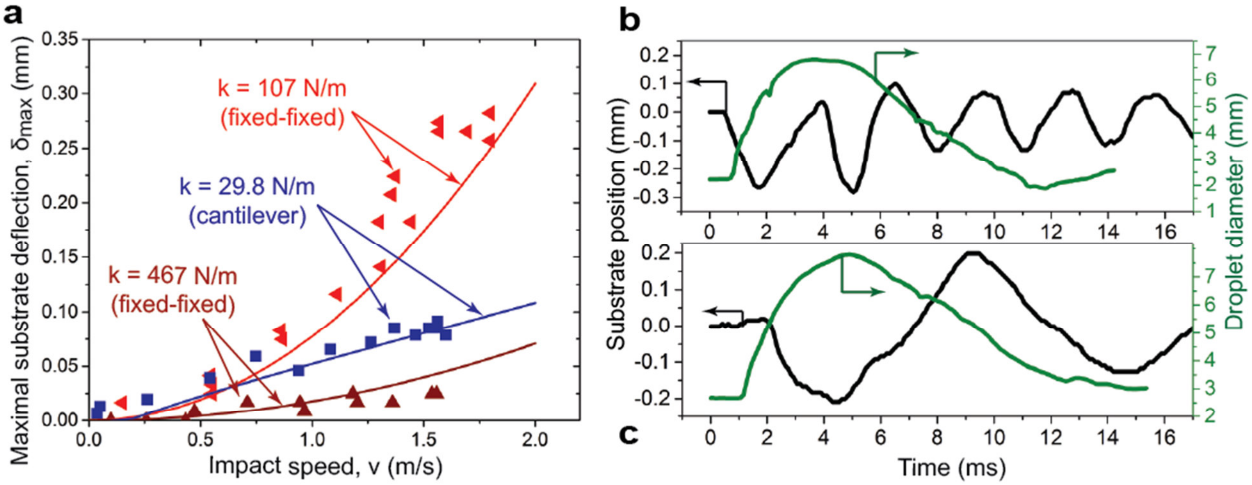

Figure S7. Substrate deflection dynamics. (a) Maximum membrane deflection for selected substrates as a function of impact speed with $\delta_{max} \sim v^2$. Shown are the deflection data and models (lines) for the fixed-fixed substrate with k = 467 N/m and $D_0 \approx 1.5$ mm, fixed-fixed with k = 107 N/m and $D_0 \approx 2.3$ mm and the cantilever style substrate with k = 29.8 N/m and a $D_0 \approx 1.5$ mm. Deflections for both fixed-fixed and cantilever style substrates can be well estimated with $\delta_{\max} = 2\frac{F_0}{k}sin(\pi\tau f_0)$, neglecting the *sin* term for fixed-fixed substrates. (b) and (c) show substrate deflection (black) and droplet diameter (green) profiles as a function of time. In (b), the substrate has a high natural frequency, and the maximum spreading of the droplet occurs near the first high point of the substrate. The substrate in (c) has a lower natural frequency, and the droplet reaches its maximum spreading near the first minimum of the substrate deflection. The maximum spreading and substrate deflection are not directly correlated and their interplay does not have an influence on the occurrence of the springboard effect.

S.9 Effects on Heat Transfer

We propose that the findings from this work can be used towards engineering new surfaces used in anti-icing and enhanced heat transfer applications. In general, shorter contact times result in smaller heat transfer rates per droplet. On anti-icing surfaces a reduction of the thermal energy transfer between solid and droplet would reduce the risk of droplet freezing[16,17].

We also propose that, when considering at many droplets hitting the surface, as for example in spray cooling, the overall average heat transfer can be increased. If the impact frequency between droplets scales with the contact time of the droplet, then a 50% decrease in contact time

could lead to a 2-fold increase in impact frequency. Assuming that the droplets can me modeled as semi-infinite bodies, then the heat transfer q scales as $\sqrt{t}$. For $f_{impact} \sim 1/t_c$, we get that the overall average heat transfer scales as $\bar{q} \sim 1/\sqrt{t_c}$, i.e. net increasing the overall average heat transfer between many droplets and a surface for reduced contact times.

References

1. Bico, J., Thiele, U. & Quéré, D. Wetting of textured surfaces. *Colloids Surf. Physicochem. Eng. Asp.* **206,** 41–46 (2002).
2. Enright, R., Miljkovic, N., Al-Obeidi, A., Thompson, C. V. & Wang, E. N. Condensation on Superhydrophobic Surfaces: The Role of Local Energy Barriers and Structure Length Scale. *Langmuir* **28,** 14424–14432 (2012).
3. Perez, M., Brechet, Y., Salvo, L., Papoular, M. & Suery, M. Oscillation of liquid drops under gravity: Influence of shape on the resonance frequency. *Europhys. Lett. EPL* **47,** 189–195 (1999).
4. Pasandideh-Fard, M., Qiao, Y. M., Chandra, S. & Mostaghimi, J. Capillary effects during droplet impact on a solid surface. *Phys. Fluids* **8,** 650 (1996).
5. Roux, D. C. D. & Cooper-White, J. J. Dynamics of water spreading on a glass surface. *J. Colloid Interface Sci.* **277,** 424–436 (2004).
6. Hung, Y.-L., Wang, M.-J., Liao, Y.-C. & Lin, S.-Y. Initial wetting velocity of droplet impact and spreading: Water on glass and parafilm. *Colloids Surf. Physicochem. Eng. Asp.* **384,** 172–179 (2011).
7. Lagubeau, G. *et al.* Spreading dynamics of drop impacts. *J. Fluid Mech.* **713,** 50–60 (2012).
8. Liu, Y., Andrew, M., Li, J., Yeomans, J. M. & Wang, Z. Symmetry breaking in drop bouncing on curved surfaces. *Nat. Commun.* **6,** 10034 (2015).
9. Mao, T., Kuhn, D. C. S. & Tran, H. Spread and rebound of liquid droplets upon impact on flat surfaces. *AIChE J.* **43,** 2169–2179 (1997).
10. Ukiwe, C. & Kwok, D. Y. On the Maximum Spreading Diameter of Impacting Droplets on Well-Prepared Solid Surfaces. *Langmuir* **21,** 666–673 (2005).
11. Clanet, C., Béguin, C., Richard, D. & Quéré, D. Maximal deformation of an impacting drop. *J. Fluid Mech.* **517,** 199–208 (2004).
12. Roisman, I. V., Rioboo, R. & Tropea, C. Normal impact of a liquid drop on a dry surface: model for spreading and receding. *Proc. R. Soc. Math. Phys. Eng. Sci.* **458,** 1411–1430 (2002).
13. Bussmann, M., Chandra, S. & Mostaghimi, J. Modeling the splash of a droplet impacting a solid surface. *Phys. Fluids* **12,** 3121 (2000).
14. Gart, S., Mates, J. E., Megaridis, C. M. & Jung, S. Droplet Impacting a Cantilever: A Leaf-Raindrop System. *Phys. Rev. Appl.* **3,** (2015).
15. Soto, D., De Larivière, A. B., Boutillon, X., Clanet, C. & Quéré, D. The force of impacting rain. *Soft Matter* **10,** 4929 (2014).
16. Shen, Y. *et al.* Icephobic/anti-icing potential of superhydrophobic Ti6Al4V surfaces with hierarchical textures. *RSC Adv* **5,** 1666–1672 (2015).
17. Ramachandran, R., Kozhukhova, M., Sobolev, K. & Nosonovsky, M. Anti-Icing Superhydrophobic Surfaces: Controlling Entropic Molecular Interactions to Design Novel Icephobic Concrete. *Entropy* **18,** 132 (2016).
